# Registered nurses’ role experiences of caring for older stroke patients: a qualitative study

**DOI:** 10.1186/s12912-021-00626-y

**Published:** 2021-06-11

**Authors:** Wei Cheng, Jiong Tu, Xiaoyan Shen

**Affiliations:** 1 Department of Pulmonary and Critical Care Medicine, Zhujiang Hospital, Southern Medical University, 510282 Guangzhou, China; 2 School of Sociology and Anthropology, Sun Yat-sen University, 510275 Guangzhou, China

**Keywords:** China, geriatric nursing, nursing care, qualitative research, role perception, stroke, working experience

## Abstract

**Background:**

With China’s population ageing rapidly, stroke is becoming one of the major public health problems. Nurses are indispensable for caring for older patients with acute and convalescent stroke, and their working experiences are directly linked to the quality of care provided. The study aims to investigate registered nurses’ experiences of caring for older stroke patients.

**Methods:**

A qualitative descriptive design was adopted. Data were collected via semi-structured interviews with 26 registered nurses about their lived experiences of caring for older stroke patients. Thematic analysis was used to analyze the data.

**Results:**

Two main themes were identified. First, the nurses identified an obvious gap between their ideal role in elderly care and their actual practice. The unsatisfactory reality was linked to the practical difficulties they encountered in their working environment. Second, the nurses expressed conflicting feelings about caring for older stroke patients, displaying a sense of accomplishment, indifference, annoyance, and sympathy. Caring for older stroke patients also affects nurses psychologically and physically. The nurses were clear about their own roles and tried their best to meet the elderly people’s needs, yet they lack time and knowledge about caring for older stroke patients. The factors influencing their working experiences extend beyond the personal domain and are linked to the wider working environment.

**Conclusions:**

Sustaining the nursing workforce and improving their working experiences are essential to meet the care needs of older people. Understanding nurses’ lived working experiences is the first step. At the individual level, nurse mangers should promote empathy, relieve anxiety about aging, and improve the job satisfaction and morale of nurses. At the institutional level, policymakers should make efforts to improve the nursing clinical practice environment, increase the geriatric nursing education and training, achieve a proper skill mix of the health workforce, and overall attract, prepare and sustain nurses regarding caring for older people in a rapidly aging society.

**Supplementary Information:**

The online version contains supplementary material available at 10.1186/s12912-021-00626-y.

## Background

Strokes are the second leading cause of death worldwide [1, 2]. In 2013, 640 million people died of a stroke [3]. With China’s population ageing rapidly, strokes are quickly becoming a major public health problem. According to a national survey conducted in 2014, the prevalence of strokes in China was 2.06 %, with about 10.36 million stroke patients over 40 years of age [4]. In particular, the incidence of stroke in people over 75 years is five to eight times higher than that in the 45–54 age group [5]. Thus, the elderly population represents the largest group of stroke patients. Stroke survivors are left with movement disorders, cognitive impairment, self-care disability, and social and communication disabilities, with some even suffering from depression and personality changes [6]. The long recovery process following a stroke requires high quality nursing care, especially for elderly people, who become more dependent on others in line with the aging process.

Nurses are indispensable in caring for patients with acute and convalescent stroke [7] and play an important role in implementing holistic nursing [8]. In hyperacute stroke units, nurses play a positive role in optimizing the thrombolytic processes, ensuring effective team communication, supporting the patients and doctors, monitoring thrombolytic complications, and improving the patient care experience [9]. However, the role of nurses in caring for older stroke patients can be challenging. Nurses may experience ambiguity regarding their role in patient care, and some feel that they lack appropriate training and fall short of providing effective care quality monitoring when caring for older stroke patients [10]. In addition, nurses find it difficult to help patients to overcome their difficulties related to performing daily tasks [11]. Their experiences, therefore, can affect the quality of care provided to these patients [12].

Although the nurses’ perspectives on caring for patients with stroke have received some academic attention [9], there is a dearth of studies focusing on nurses’ perspectives and experiences in caring for older stroke patients, especially in the context of China, with its rising number of older patients. Nurses’ attitudes toward older people affect the quality of care provided to the aging population. Earlier surveys among Chinese nursing students in 2010 and 2012 found the majority were not willing to care for older people [13] and working with older people was ranked as one of the least preferred areas [14]. More recent researches revealed a change in attitudes, i.e., nursing students’ expectancy and motivation for choosing gerontological nursing as a career were at a moderate level [15], their care willingness of elderly was in medium-high level [16], and registered nurses also held a slightly positive attitude toward older adults [17]. These existing researches showed a diverse and changing picture of nurses’ attitudes toward caring older people in China, yet most of these researches were quantitative in nature, unable to capture the nuanced picture of what nurses actually think and feel.

Therefore, we conducted a qualitative study to explore the lived experiences of nurses in caring for older stroke patients on neurology wards. Understanding such lived experiences can facilitate the discussion of how to develop humane management measures for neurological nursing staff as well as improve the quality of care provided for older patients. Besides, nurses’ experiences of caring for older stroke patients can inspire us to understand the current situation and challenges within geriatric nursing, in order to prepare more effectively to meet the needs of the rapidly aging society.

## Methods

### Design

A qualitative descriptive design was adopted in this study to explore the working experiences of the nurses. The qualitative descriptive study is concerned with understanding the individual human experience in its unique context [18], it fits when straightforward descriptions of experiences and perceptions are desired [19]. In other words, this design is suitable to explore nurses’ perceptions and experiences of caring older people in their own social context.

### Setting and participants

This study was conducted in the department of neurology of a grade-III level-A hospital (one of the highest-ranking hospitals) in Guangzhou, China. In this 144-bed stroke screening center, more than 40 % of the patients are elderly and about 80 % of them are stroke patients. In total, there are 64 nurses in the department, among whom 26 nurses accepted our invitation to attend an interview. The interviewed nurses were aged 24–53 years old, with an average age of 28.15 years. They had 1–30 years of experience of caring for older stroke patients, with an average working experience of 6.04 years (see Table 1). All of the participants were informed of the aim and design of the study. Informed consent was obtained from all of the participants prior to the interview. Ethical approval was obtained from the Ethics Committee of the author’s institution.

**Table 1:** Characteristics of the nurses interviewed (*n* = 26)

Characteristics	N (%)
**Age**	
20–29	19 (73.1)
30–39	5 (19.2)
≥40	2 (7.7)
**Gender**	
Female	26 (100 %)
**Years in practice**	
5<	13 (50)
5–10	9 (34.6)
> 10	4 (15.4)

### Data collection

The authors conducted one-on-one interviews with the participants. The interviews were semi-structured, centering on the nurses’ perceptions and experiences of providing care to older stroke patients. Some main questions were asked to illicit the interviewees’ views, such as: What role do you think nurses play in caring for older stroke patients? How do you care for older stroke patients? What affects you in the process of caring for older stroke patients? (See the Interview Guide in the Appendix.) The interviewers posed follow-up questions in response to what the interviewees said; for example, “Why do you have these feelings?”, “For example?” and “Can you please describe that in detail?” Each interview lasted between 30 min and one hour. All of the interviews were recorded and transcribed verbatim within 24 h of the data collection, to ensure accuracy. We interviewed to saturation, when we found that the viewpoints that the interviewees were expressing were repeated and no new points were emerging [20].

### Data analysis

In qualitative descriptive research, content and thematic analyses are the most commonly used data analysis techniques, yet compared to content analysis that allows the researchers to analyse data qualitatively as well as quantify the data, thematic analysis provides a purely qualitative account of the data that is richer and more detailed [18]. In this study we used thematic analysis to identify and report themes within the interview transcripts [21], in order to provide a rich thematic description of the experiences of nurses related to caring for older stroke patients. The first two authors coded the same transcript independently. The researchers read and re-read the transcripts to grasp the overall information, and codes were allocated to relevant narratives in the process. All of the codes were further consolidated into categories and more comprehensive themes, the researchers then compared and discussed them in order to reach agreement. Overall, two main themes and seven subthemes were identified regarding the nurses’ experiences of caring for older stroke patients (see Table 2).

**Table 2:** Main Themes and Subthemes

**Themes**	**Subthemes**	**Explanation**
**Gap between ideal and reality**	**Role expectations**	Care provision
		Education and guidance
		Psychological care and social support
	**Unsatisfied reality**	Focus on treatment tasks and paperwork
		No time for bedside and psychological care
	**Practical difficulties**	Heavy workload
		Lack time
		Insufficient knowledge
	**Efforts to improve**	Do as much as possible
		Provide active services
**Conflicting feelings**	**Compassion and annoyance**	Showing compassion
		Feeling annoyed sometimes
	**Achievement and indifference**	A sense of accomplishment and contentment when patients improve
		Low spirits and a sense of futility when patients fail to make progress
		Become indifferent over time
	**Physical and psychological impact**	Fear becoming old and scared of getting sick
		Occupational diseases

### Rigor

We adopted the following measures to improve credibility, ensure accuracy and reduce bias. The first author, being a caregiver for older stroke patients, established a good rapport with the interviewees, which enabled us to acquire an in-depth understanding of the nursing experience. The participants were given the freedom to choose the date, time, and setting of their interview in order to maximize their convenience and comfort, and minimize the impact of the environment on their responses. The research credibility was further ensured through a detailed recording and presentation of the data. Moreover, the first two authors coded the data independently and compared their respective results with each other to ensure dependability and enhance accuracy. The analysis process continued until no new themes emerged and saturation had been reached [20]. All methods in this study were carried out in accordance with qualitative descriptive study and thematic analysis guidelines and regulations. The COREQ checklist was provided as attached file to further ensure methodological rigor.

## Results

### Gap between the ideal care role and actual practice

#### Role expectations

All of the interviewed nurses emphasized the importance of nursing care regarding stroke patients’ recovery. The respondents saw their work as encompassing the roles of caregiver, planner, educator, consultant, and supervisor. They mentioned that, when taking care of stroke patients, it was essential not only to provide care for the patients, but also to educate them about self-care; not only to manage the patients’ physical condition (blood pressure, blood sugar level, etc.), but also to provide psychological support and be a friend at times.

*I feel that stroke patients are in great need of guidance. For the psychological aspect, we first need to encourage him, tell him that hemiplegia does not mean relying on other people for everything; you (the patient) can still do many things on your own. In terms of falls, fall prevention does not mean staying at home and not going anywhere. You (the patient) need to know the factors that cause falls, becoming familiar with your surrounding environment, and take precautions…Guidance is essential for recovery and care.* (Interview 1)

Education and guidance are particularly important for chronic conditions like strokes, and also for older people who may become forgetful sometimes, and their family caregivers, who may lack expert knowledge. In the process, nurses should display great patience and possess sufficient knowledge. During the interviews, it was clearly felt that the nurses’ work extended beyond basic care to include psychological care, social support, and so on. Nurses are not only the executor of the doctor’s prescription but also consider the needs of the patients and their families.

*Many patients and family caregivers lack knowledge about strokes. They often feel confused and fear the occurrence of stroke-induced paralysis and other symptoms. Caregivers often do not know how to care for stroke patients, so they ask all kinds of strange questions. As nurses, we need to be more patient when communicating with them and give them appropriate guidance and psychological counseling. (Interview 23)*

#### The unsatisfactory reality

However, the nurses generally admitted that they lacked sufficient time or professional knowledge to perform their ideal nursing role, and so focused instead on what is strictly necessary. Currently, their time was taken up with treatment-related tasks and paperwork, so they spent relatively little time at the patients’ bedside. Instead, there were 12 care workers working in the neurology department. These family-paid care workers took up many bedside works that previously should be within the nurses’ scope of responsibility.

*Only when nurses carry out bedside work can high quality nursing be achieved…however, we nurses in public hospitals, especially big hospitals, focus on dispensing medicine and giving injections and infusions. Many of the patients’ bedside care is left to the family and (family-hired) care workers. (Interview 22)*

*We nurses should adopt the role of caregiver, care planner, consultant… Up to now, I have only adopted the role of caregiver. Most of our time is spent on giving treatment and doing basic nursing. We rarely have time to communicate with the patients and their families, and can provide hardly any education and consultation for the patients. (Interview 18)*

Albeit recognizing the importance of humane and psychological care, the nurses were not sure exactly how to provide it. Few of them had received any psychological training and they did not have time to undertake extra caring work.

*Their psychological needs should be one of the major aspects, yet we haven’t done that. For the information (aspect), patients and their families certainly want to know more about the illness, so sometime they ask us nurses. If the nurse is experienced, with a good attitude, she can tell them clearly in detail. However, in most cases, we are too busy (to communicate for long) and some (junior nurses) lack sufficient expert knowledge to explain (clearly) to the patients, so they are still left confused (Interview 2)*.

#### The practical difficulties encountered

The above unsatisfactory nursing practice, according to our interviewees, was related to their working environment, such as the nursing shortage, lack of nursing time, labour-intensive workload, and the need for knowledge acquisition. The hospital wards were full of patients, sometimes with additional beds for new patients that exceeded the usual bed capacity.

*We have many patients. My (nursing) team care for patients with relatively light symptoms, yet there are about 30 patients. Sometime I work on the afternoon shift on my own. There are many drips to give, and new patients are coming in (need to be registered). It seems that there are endless tasks to do, and we are always short of time. (Interview 12)*

Although there were over 60 nurses in the neurology department, these nurses were divided into three shifts, and further into team groups for each shift, which meant that one or two nurses per team took care of dozens of patients. Moreover, there was a lot of paperwork to complete.

*There are 64 nurses in my department, which is already more than (many) other departments. Yet (psychological nursing and other humane care) cannot be offered. We want to spend (our nursing) time on the patients yet there are so many evaluation sheets and forms to fill in. We work for eight hours a shift, except for one hour for a meal, which leaves only seven hours. A patient’s paperwork takes about 20 min. If five, six (new) patients are enrolled, it takes hours to fill in the forms, so how can I do anything else? (Interview 1)*

Nurses generally have heavy workload and frequently work overtime. There are significant gaps between high-intensity and high-quality nursing. Moreover, the nurses stated that some of them lack knowledge and training about geriatric nursing in general and strokes in particular.

*Regarding providing health education (for patients and their families), I feel that I haven’t done enough. Why? A lack of knowledge! When I was at nursing school, what we learned about health education was quite simple and repetitive. When I worked in the neurology department, I found there was a lot of specialized knowledge to teach the patients (and their families); for instance, how to swallow and drink to avoid choking and coughing, how to care for patients with physical malfunctions, how to turn (a bedridden patient) over. I was not taught this specialized knowledge and have to gain experience slowly while in work. (Interview 8)*

#### Efforts to improve

Albeit encountering various barriers to providing ideal care, the nurses whom we interviewed generally expressed a willingness to improve and many tried their best to take care of patients as far as their time and capacity allowed.

*(I try to) be patient and gentle. Because older people may not understand what I say, I try to speak slowly, repeat what I’ve said a few times, try to have good attitude. (Interview 12)*

Once the nurses had finished their allocated tasks related to treatment, basic nursing and paperwork, they sometimes also took the initiative to communicate with stroke patients and their caregivers, inquired about the patients’ care needs, soothed their worries about the illness, and prudently provided health education to the patients and their families. They also sought the help of doctors if they were unsure of the accuracy of the information to be provided.

*I try to have a chat with them and tell the patients and caregivers not to be nervous about the disease. I encourage them by saying that a lot of patients who have suffered from a similar illness have had good outcomes. If they require professional knowledge about the illness, I try to provide them with as much knowledge as I can. Sometimes, I worry about the fact that maybe what I say will conflict with the doctors’ opinions, so I advise them to ask the doctor directly, or promise to help them to consult a doctor. (Interview 20)*

### Conflicting feelings about caring for older stroke patients

Faced with the above-mentioned gaps between their ideal role and the unsatisfactory reality, the nurses expressed conflicting feelings about their work and the patients for whom they cared.

#### Showing compassion and feeling annoyed

In terms of the nurses’ attitudes toward the patients, the frail older patients often reminded the nurses of their own grandparents or children who needed care and compassion. Controlling the symptoms and complications associated with strokes also require nurses to devote a lot of attention to these patients. Many nurses were very sympathetic about the older people’s plight and made their best effort to care for them.

*I feel they are like my grandparents, are ordinary older people. Sometimes, I can’t help ‘nagging’ them, telling them again and again what they should be cautious about, how to take their medicine, how to eat and drink, in case they make mistakes. It’s just like the way I tell my grandparents. (Interview 13)*

*Older people, like little kids, need to be cared for and comforted. (Interview 17)*

Yet, after working a long shift on the ward, the nurses may feel annoyed sometimes. The nurses were the direct providers of health services and the first point of contact for many patients and their families. Our interviewees reported that the patients and their families frequently expected them to answer all kinds of enquires, and that they were often asked to do things that were beyond their ability and scope of responsibility; therefore, they felt annoyed and sometimes offended.

*In the beginning, I liked older people very much and loved this profession but, as time goes by, facing the same illness every day and handling the same (physical) problem, I get bored… I’m not bothered by the work itself, but there are things that are just beyond our ability, for instance, they ask me about medical bills, complain about the hospital environment, ask why the doctor hasn’t arrived, and I become annoyed. However, if they ask me about what to eat or do in their current state, I wouldn’t be bothered at all. I’m OK with the nursing care itself. (Interview 7)*

*Older people do not have a good memory; they ask a lot, and ask the same question repetitively. Some older people are eccentric or stubborn. Some may easily become anxious; some even have the symptoms of depression. We (nurses) may certainly feel annoyed sometimes. (Interview 25)*

#### Sense of achievement and indifference

In terms of their attitudes toward their work, the nurses reported varied experiences and perceptions related to caring for older stroke patients. The patients undergo a long recovery process after stroke. Most nurses expressed a sense of accomplishment and contentment when they saw a patient’s condition improve.

*When I see the patients under my care getting better day by day, I feel a sense of achievement. (Interview 4)*

*For the patients where the treatment works well, I feel very good. When they were first hospitalized, they had all kinds of tubes and drains on the body, couldn’t get out of bed at all. Now they talk to us, tell jokes, and practice walking again with their family’s help. Watching them, I feel that what I do is worthwhile. (For those that do not recover well?) Sometimes a feeling of indifference. (Interview 2)*

While some stroke patients may recover well, bringing nurses a sense of worth, other older stroke patients may lie in the bed for a long time, making little progress. In these cases, the nurses may become low spirited and feel worthless.

*Previously, there was a patient with a brain stem hemorrhage who was in a coma and had been having sporadic bouts of fever ever since he was admitted to the intensive care unit. There were no signs of him waking up…we always have a feeling of indifference when we care for a patient like him. They cannot speak or move and cannot cooperate with us.* (Interview 2)

*Stroke patients may be admitted to the hospital again and again, never completely recovering. Some even have more than one stroke, and their condition deteriorates over time. I have got used (to their unchanged condition), and do not have much feeling. (Interview 6)*

The nurses reported often experiencing negative and dispirited reactions when they cared for older patients with poor outcomes. The words they mentioned most were “indifference”, “numbness”, and “tiredness”. They can hardly sustain their compassion and gradually become indifferent over time. Indifference is also a survival strategy for them to counter job burnout, for they receive insufficient incentives regarding their work.

*When I first joined the department, I was passionate, hoping to really do something but the department has a heavy workload but a relatively low income so, as time goes by, I feel tired, really tired. (Interview 23)*

#### The impact on the nurses of caring for older people

According to the nurses interviewed, caring for older patients had an impact on them, both physically and psychologically. It makes them scared of becoming sick and old. Moreover, owing to their heavy workload and labor-intensive work, they are prone to suffering from lower back pain, lumbar disease, and other occupational diseases. Consequently, the nurses reported feeling that they were ageing rapidly, both physically and psychologically.

*Older people are unlike children who become revitalized after treatment. As I have been working with them for long, I find myself getting old too both psychologically and physically. The workload is so heavy and labor-intensive. You see, I now have lumbar muscle strain and cervical disease. If there were other jobs available (for me) I might quit. (Interview 7)*

*This (neurology) department is the most dirty and tiresome department in this hospital. I feel bored after working here for five, six years. Sometimes, I feel I can’t stay here anymore…Although I am in my twenties, I feel I am already over 30 psychologically. (Interview 1)*

Watching older people who are bed-ridden due to a stroke make little progress, the nurses often consider their own health and future life and career. They are scared of being in a similar condition when they are old and expressed a sense of uncertainty about the future.

*Oh, don’t get a disease like this (in old age). It’s a real trouble (an expression of fear). (Interview 9)*

*I feel we should give older people more care and concern but, probably, our current job is too busy or we have got used to it, and the concern and care for older people are far from enough. Sometimes, I worry about what I’ll do when I become old myself. (Interview 11)*

## Discussion

In this study, the nurses narrated their role perceptions and experiences related to caring for older stroke patients. There is an obvious gap between the ideal role and their actual nursing practice. The nurses perceive their ideal role as involving a variety of responsibilities in order to meet the care needs of the patients and their families. This is, in part, consistent with the previous research, which suggested that nurses should play multiple roles in relation to stroke patients, including caring for patients, interpreting the patients’ conditions, and comforting anxious patients [22]. The ideal professional nursing role is that of a caregiver, educator, manager, and supervisor [23].

However, the nurses in this study seem to find it more difficult to achieve their ideal role compared with their counterparts in western countries. They expressed their sense of helplessness and frustration at work, including a lack of professional knowledge and a shortage of time and human resources. These factors left them unable to attend to their patients’ psychological needs and provide sufficient care. Instead, in many public hospitals, it is the family-paid care workers (*hugong*) providing most bedside care. These care workers aid patients as well as nurses, taking up many responsibilities that previously should be within the nurses’ scope of responsibility, yet they cannot guarantee the quality and expertise of the care services provided [24, 25]. Our findings from the nurses’ perspectives also echo other research conducted in China from the patients’ perspective, which indicates that a gap exists between the nurses and patients regarding the understanding of care and that the patients required more support from the nurses regarding their psychological needs [26], so the nurses’ perception of their patient’s quality of life may not accurately reflect the older people’s actual experiences [27].

Our study further indicates that the gaps between the ideal and the reality impacted on the nurses’ feelings about caring for older stroke patients. The nurses expressed seemingly conflicting feelings: a sense of accomplishment, indifference, annoyance, and sympathy. Their emotional experiences related to nursing older stroke patients vary dynamically, influenced by the stroke severity and prognosis. Moreover, when nurses are unable to meet the needs of older stroke patients because of the limited nursing resources, they may experience negative emotions. In addition, the participants described the impact of caring for older people on them, both psychologically and physiologically. They were psychological ageing because of the constant exposure to the frail elderly, and scared of developing a similar illness themselves in old age. The nurses also reported physiological ageing in terms of occupational diseases resulting from their work. Occupational diseases, such as lower back pain, were also widely reported among nurses in other countries [28].

The nurses made efforts to bridge the gap between their ideal role and the reality. They stated that, when patients and their families had many questions about strokes, they tried their best to provide explanations and offer psychological counseling. Yet, some of the obstacles proved too great for them, as the factors influencing their work extend beyond the personal domain and are linked to the wider working environment. Chinese public hospitals are overcrowded with patients, yet there is a serious shortage of nursing staff [29]. Nurses frequently work overtime, burdened by too many tasks, which causes job burnout [30] and influences the quality of care delivered. The conflicting feelings that the nurses experienced at work were a reflection of their working environment. They were also consistent with the finding in the previous literature that nurses in China experienced a low level of job satisfaction and a high level of turnover intention in their current working environment [31, 32]. Besides, despite the fact that China is facing a rapidly aging population, Chinese gerontological nursing is relatively new, and yet to achieve the international standards [27]. The lack of professional knowledge and competencies (in both geriatric nursing and stroke care) is one of the factors preventing nurses from playing their ideal nursing role, but knowledge alone is insufficient to develop expertise, as motivation and attitude also play an essential role [33]. The nurses in this study reported having low job satisfaction and morale regarding caring for older stroke patients, which may impact on their willingness to remain in the nursing profession. They need to be motivated and regenerated in their work when facing the long recovery process of older stroke patients.

### Implications and recommendations

An understanding of nurses’ experiences and perceptions is necessary to improve the care provided to older stroke patients. At the individual level, nurses should have more training on how to handle conflicting feelings, obtain more knowledge on geriatric nursing, and receive quality improvement education and continued training to improve their professional capabilities. Moreover, their working spirit needs to be regularly re-generated to inspire them to take care of older people, whose condition may not improve in the same way as younger people. Furthermore, nurse managers should be aware of the psychological and physical impact of caring for older people, revitalize nurses’ positive experiences and feelings to improve their job satisfaction, and plan preventive and curative measures to reduce the prevalence of occupational diseases among nursing personnel.

At the institutional level, changes are needed to facilitate the nurses’ work, such as easing nurses’ working schedule and workload, matching their heavy workload with a proper income, offering guidelines and adequate information at the right time to support nurses engaged in geriatric practice, and providing a working environment that is in line with the holistic role of nurses and policies to enable holistic nursing. With an aging population that creates rising needs of health care, hospitals should achieve a proper skill mix of the health workforce according to the care needs of the older patients, for instance, not only incorporating but also training assistant personnel in the health workforce. Overall, due to the increasing number of older people, it is urgent and necessary to sustain the nursing workforce in geriatric care more effectively, and change the overall circumstances of nurses and the shortage of nurses as a whole.

### Limitations

The present study had a few limitations. The research was based in the neurology department of a top-level hospital, so the working experiences of the nurses there may differ from those of nurses working in other institutions. Moreover, we did not consider the nurses’ demographic and job characteristics (such as age, years of practice, job position, and gender), albeit recognizing that these variables may impact on the nurses’ working experiences and perceptions. For instance, all of our interviewees were female, yet there were four male nurses working in the neurology department, who were unable to take part in the interviews. The results may have been different had these male nurses been included. Nevertheless, a greater understanding of nurses’ general experiences and perceptions of caring for older people will inspire policymakers to envision targeted measures to improve nurses’ job satisfaction as well as the quality of care provided.

## Conclusions

Against a background of rapid population aging in China, sustaining the nursing workforce and improving nurses’ working experiences are essential to meet the care needs of older people. Our results highlight nurses’ subjective experiences related to caring for older stroke patients: there is an obvious gap between the nurses’ ideal role and their actual practice, and they have conflicting feelings about caring for older stroke patients, which were affected by the stroke severity of the patients as well as factors within their working environment. Caring for older stroke patients also affects nurses psychologically and physically. The nurses were clear about their own role and tried their best to meet the elderly’s need, yet some of the challenges were linked to the wider working environment and difficult for them to overcome, hence many more changes and support are needed at the institutional level.

## Supplementary information

**Additional file 1.**

## Data Availability

Data is available upon reasonable request from the corresponding author.
