# Supplementary material for: Registered nurses’ role experiences of caring for older stroke patients: a qualitative study
Source: BMC Nurs. 2021 Jun 11;20:96. doi: 10.1186/s12912-021-00626-y (PMC8196525; doi:10.1186/s12912-021-00626-y)
Supplement: Supplementary file 1 — Additional file 1. [file 12912_2021_626_MOESM1_ESM.docx]

# Registered nurses’ role experiences of caring for older stroke patients: A qualitative study

Wei Cheng^1^, MD, RN; Jiong TU^2^, PHD; Xiaoyan Shen^1^, MS, RN

1. Zhujiang Hospital, Southern Medical University, Guangzhou, 510282, China.

2. School of Sociology and Anthropology, Sun Yat-sen University, Guangzhou, 510275, China

§ Wei Cheng and Jiong Tu contributed equally.

**Corresponding author:** Jiong Tu, The School of Sociology and Anthropology, Sun Yat-sen University, Guangzhou, 510275, China. Email: [tujiongnk@gmail.com](mailto:tujiongnk@gmail.com)

## Appendix: Interview Guide

1. Patients in neurology wards mostly suffer from stroke, and most of them are older patients. How you think about caring for older people, compared to younger patients?
2. What role do you think nurses play in caring for older stroke patients?
3. How do you care for older stroke patients in practice? Is there any special experience in caring for older stroke patients?
4. How you feel in the process of caring for older stroke patients?
5. What really impacts/affects you in the process of caring for older stroke patients?
6. How the experiences of caring for older stroke patients affect you (both negatively and positively)?
7. Is there any improvement needed? What and how?
8. Is the neurological department your first choice? Why?
9. Anything else you feel important in your work?
